# KCC2 is required for the survival of mature neurons but not for their development

**DOI:** 10.1016/j.jbc.2021.100364

**Published:** 2021-02-02

**Authors:** Georgina Kontou, Shu Fun Josephine Ng, Ross A. Cardarelli, Jack H. Howden, Catherine Choi, Qiu Ren, Miguel A. Rodriguez Santos, Christopher E. Bope, Jake S. Dengler, Matt R. Kelley, Paul A. Davies, Josef T. Kittler, Nicholas J. Brandon, Stephen J. Moss, Joshua L. Smalley

**Affiliations:** 1 AstraZeneca-Tufts Laboratory of Basic and Translational Neuroscience, Tufts University School of Medicine, Boston, Massachusetts, USA; 2 Department of Neuroscience, Tufts University School of Medicine, Boston, Massachusetts, USA; 3 Department of Neuroscience, Physiology, and Pharmacology, University College London, London, UK; 4 Neuroscience, IMED Biotech Unit, AstraZeneca, Boston, Massachusetts, USA

**Keywords:** KCC2, apoptosis, extrinsic pathway, cell death, seizures, AAV, adeno-associated virus, DIV, days *in vitro*, GABA_A_R, γ-aminobutyric acid type A receptors, GFP, green fluorescent protein, KCC2, K^+^/Cl^−^ cotransporter 2, KO, knockout, PARP, poly (ADP-ribose) polymerase (PARP), TLE, temporal lobe epilepsy, TTX, tetrodotoxin

## Abstract

The K^+^/Cl^−^ cotransporter KCC2 (*SLC12A5*) allows mature neurons in the CNS to maintain low intracellular Cl^−^ levels that are critical in mediating fast hyperpolarizing synaptic inhibition *via* type A γ-aminobutyric acid receptors (GABA_A_Rs). In accordance with this, compromised KCC2 activity results in seizures, but whether such deficits directly contribute to the subsequent changes in neuronal structure and viability that lead to epileptogenesis remains to be assessed. Canonical hyperpolarizing GABA_A_R currents develop postnatally, which reflect a progressive increase in KCC2 expression levels and activity. To investigate the role that KCC2 plays in regulating neuronal viability and architecture, we have conditionally ablated KCC2 expression in developing and mature neurons. Decreasing KCC2 expression in mature neurons resulted in the rapid activation of the extrinsic apoptotic pathway. Intriguingly, direct pharmacological inhibition of KCC2 in mature neurons was sufficient to rapidly induce apoptosis, an effect that was not abrogated *via* blockade of neuronal depolarization using tetrodotoxin (TTX). In contrast, ablating KCC2 expression in immature neurons had no discernable effects on their subsequent development, arborization, or dendritic structure. However, removing KCC2 in immature neurons was sufficient to ablate the subsequent postnatal development of hyperpolarizing GABA_A_R currents. Collectively, our results demonstrate that KCC2 plays a critical role in neuronal survival by limiting apoptosis, and mature neurons are highly sensitive to the loss of KCC2 function. In contrast, KCC2 appears to play a minimal role in mediating neuronal development or architecture.

The K^+^/Cl^−^ cotransporter KCC2 (encoded by the gene *SLC12A5*) is the principal Cl^−^-extrusion mechanism employed by mature neurons in the CNS (1). Accordingly, its activity is an essential determinant for the efficacy of fast synaptic inhibition mediated by type A γ-aminobutyric acid receptors (GABA_A_R), which are Cl^−^ permeable ligand-gated ion channels. At prenatal and early postnatal stages in rodents, neurons have elevated intracellular Cl^−^ levels resulting in depolarizing GABA_A_R-mediated currents (2). The postnatal development of canonical hyperpolarizing GABA_A_R currents is a reflection of the progressive decrease of intraneuronal Cl^−^ that is caused by the upregulation of KCC2 expression and activity, which do not reach their maximal levels in humans until 20 to 25 years of age (3). Consistent with this critical role in regulating inhibitory neurotransmission, patients with mutations in *SLC12A5* exhibit severe epilepsy that develops after birth, together with severe developmental delay (4, 5, 6). The essential role KCC2 plays in the brain is further exemplified by gene knockout studies in mice where homozygotes die shortly after birth (7, 8). In addition to neuronal Cl^−^ homeostasis, KCC2 has been proposed to play a role in determining the morphology of dendritic spines that is independent of its Cl^−^ transport function (9, 10, 11).

Alterations in KCC2 function have been reported in patients with temporal lobe epilepsy (TLE) (12, 13, 14). In addition to spontaneous seizures, patients with TLE have profound changes in the structure of the hippocampus, which include extensive neuronal death, modified arborization of surviving neurons, and reactive gliosis. KCC2 activity is modulated by phosphorylation at multiple sites, and altering KCC2 phosphorylation has also been shown to modify the development and severity of seizures (15, 16, 17). Significantly, we have recently shown that local inactivation of KCC2 in the hippocampus is sufficient to induce the development of spontaneous seizures resembling those seen in TLE (18) and a marked increase in neuronal death. However, the direct contribution of KCC2 expression levels and/or activity to the neuronal loss and anatomical changes seen in TLE is unknown.

To investigate the role that KCC2 plays in determining neuronal viability, we have examined the consequences of ablating its expression prior to and following neuronal development. In mature cultured neurons and the adult hippocampus, decreasing KCC2 expression activated the extrinsic apoptotic pathway resulting in rapid neuronal death. Acute pharmacological blockade of KCC2 also induced apoptosis by a similar mechanism. Ablating KCC2 expression levels in immature neurons compromised the development of hyperpolarizing GABA_A_R currents but did not impact on neuronal viability, proximal dendritic arborization, or spine formation. Collectively our results suggest that KCC2 is required for survival of mature neurons and hyperpolarizing GABA_A_R currents but is dispensable for their development and morphology.

## Results

### Confirming Cre-dependent ablation of KCC2 expression in mature and immature neurons

**Figure 1:**
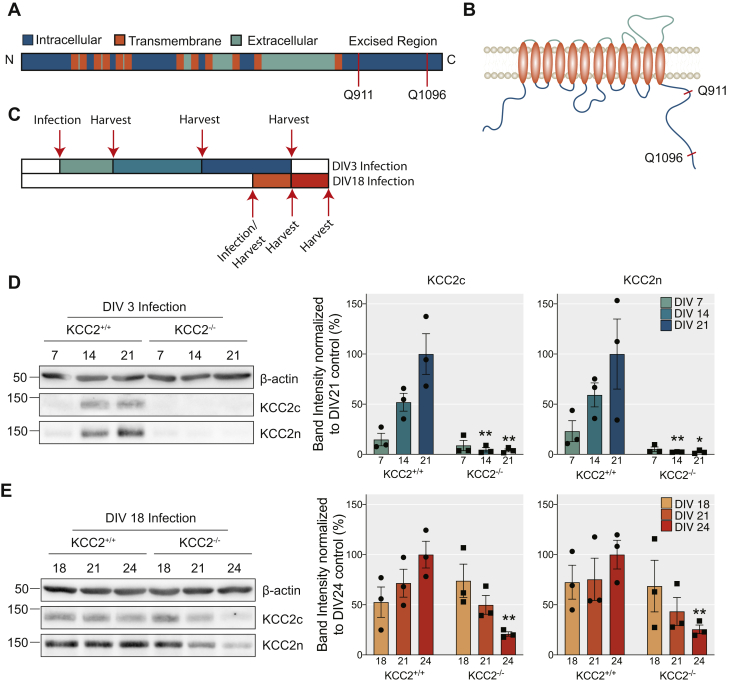
**Using a viral approach to knock out KCC2 from mature and developing dissociated cortical/hippocampal neurons.***A*, schematic diagram of the KCC2 gene, denoting the region excised by the Cre-recombinase. *B*, schematic diagram of KCC2 protein topology, denoting the excised region on the intracellular C terminus. *C*, schematic diagram of viral treatment paradigm to knock out KCC2 in developing (DIV3) and mature (DIV18) neuronal networks. *D*, immunoblots and corresponding *bar chart* quantification confirming the reduction of KCC2 expression after AAV-Cre viral infection of KCC2^fl^ cortical/hippocampal dissociated neurons at DIV3 (n = 3 replicates). *E*, immunoblots and corresponding *bar chart* quantification confirming the reduction of KCC2 expression after AAV-Cre viral infection of KCC2^fl^ cortical/hippocampal dissociated neurons at DIV18 (n = 3 replicates).

In order to investigate the significance of KCC2 for neuronal development, maintenance, and viability, we generated mixed cortical/hippocampal cultures from P0 mice in which *loxP* sites have been introduced into the *SLC12A5* gene (KCC2^fl^). These cultures were then infected with adeno-associated viruses (AAVs) *AAV9-CaMKII-eGFP* (AAV-GFP), or *AAV9-CaMKII-eGFP-Cre* (AAV-Cre). Using the CaMKII promoter, we were able to target Cre expression to principal neurons after 3 (immature) or 18 (mature) days *in vitro* (DIV) (Fig. 1, *A*–*C*). The expression of Cre recombinase leads to the excision of exons 22 to 25 between the two *loxP* sites inserted into the *SLC12A5* gene (Fig. 1*A*) resulting in a translated protein that lacks amino acids Q911-Q1096 of KCC2 that are essential for transporter activity (18, 19) (Fig. 1*B*).

To determine the efficiency of recombination, immature neurons infected at DIV3 were harvested at DIV7, DIV14, and DIV21, lysed, and immunoblotted with KCC2 antibodies (Fig. 1*C*). Using a C-terminal antibody, KCC2 expression levels increased over this time course in neurons infected with AAV-GFP. In neurons infected with AAV-Cre, KCC2 levels were reduced to 4.5 ± 2.18% and 3.9 ± 1.46% at DIV14 and 21 respectively compared with control (DIV14: *p* = 0.007, DIV21: *p* = 0.009, unpaired *t*-test) (Fig. 1*D*). Using an N-terminal antibody, KCC2 expression levels were also reduced significantly in cultures infected with AAV-Cre (DIV14: 2.3 ± 0.14%, *p* = 0.009, DIV21: 2.4 ± 0.99%, *p* = 0.049, unpaired *t*-test). The residual KCC2 observed is likely to be that expressed in GABAergic interneurons. To assess the efficiency of KCC2 removal in mature networks, we harvested neurons for western blot analysis on the day of (DIV18), 3 days (DIV21), and 6 days (DIV24) after viral infection (Fig. 1*C*). The total protein levels of KCC2 were gradually reduced 3 and 6 days after infection (Fig. 1*E*). By DIV24, KCC2 levels were reduced to 20.9 ± 2.21% (*p* = 0.004, unpaired *t*-test) and 25.5 ± 4.30% (*p* = 0.008, unpaired *t*-test) in the AAV-Cre compared with the control, as detected by the C- and N-targeting antibodies respectively. Collectively these results demonstrate that viral infection using AAV-Cre is an efficient strategy to reduce KCC2 expression levels in developing and mature neuronal networks.

### Reducing KCC2 expression does not modify neuronal arborization proximal to the soma

**Figure 2:**
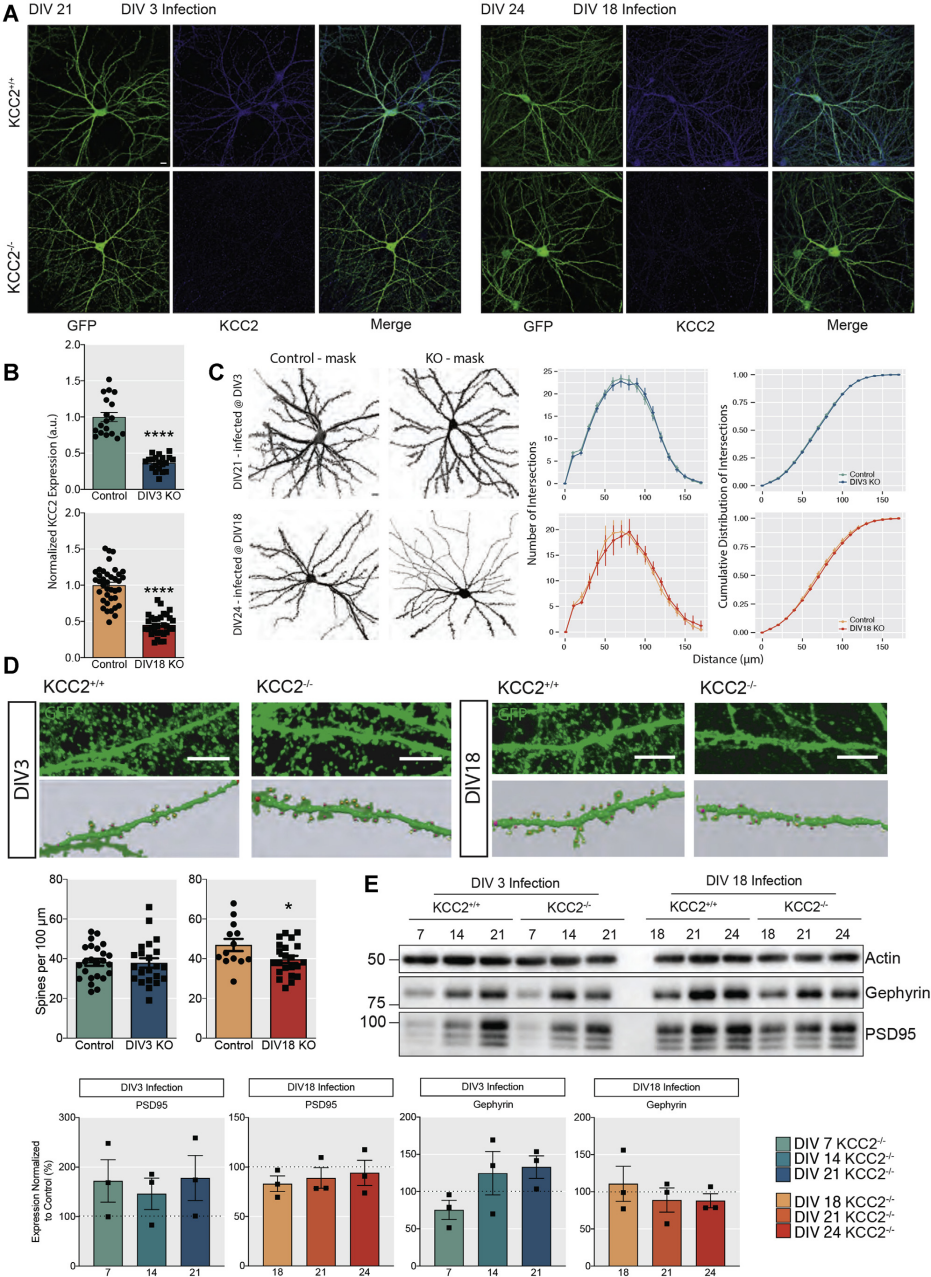
**The effect of removing KCC2 from developing and mature networks on maintenance of dendrites, spines, and synapses.***A*, 1024 × 1024 max projected confocal images of primary cultured neurons from P0 pups infected with AAV-GFP or AAV-Cre at DIV3 and DIV18 and fixed at DIV21 and DIV24, respectively. The cells were immunostained for KCC2 and GFP to amplify the signal, and confocal images were acquired using a 60× objective (Scale bar = 10 μm). *B*, quantification of the normalized mean fluorescent KCC2 signal within GFP-positive neurons for DIV3-infected and DIV18-infected cells. (DIV3: n_KCC2+/+_ = 18, n_KCC2−/−_ = 20 neurons, DIV18: n_KCC2+/+_ = 16, n_KCC2−/−_ = 16 neurons from 4 to 6 individually infected cultures). *C*, neuronal reconstruction of control and knockout neurons infected with the AAV-GFP or AAV-Cre for Sholl analysis using the Simple Neurite Tracer Fiji plugin. The number of intersections (y-axis) are plotted against distance from the soma (x-axis) and as a cumulative distribution. (DIV3: n_KCC2+/+_ = 22, n_KCC2−/−_ = 19 neurons, DIV18: n_KCC2+/+_ = 9, n_KCC2−/−_ = 9 neurons from 4 to 6 individually infected cultures) (Scale bar = 10 μm). *D*, cropped confocal images of GFP-expressing neuronal processes with dendritic spines (Scale Bar = 10 μm) with spine reconstructions below, made using Neuron Studio (48). Quantification of the number of spines per 100 μm of dendritic process (DIV3: n_KCC2+/+_ = 24, n_KCC2−/−_ = 22 dendritic segments, DIV18: n_KCC2+/+_ = 13, n_KCC2−/−_ = 22 dendritic segments from 4 to 6 individually infected cultures). *E*, immunoblots showing the levels of the excitatory postsynaptic marker PSD95 and the inhibitory postsynaptic marker gephyrin in AAV-GFP and AAV-Cre infected cells at DIV3 and DIV18 (n = 3 replicates).

To assess the effects of reduced KCC2 expression on neuronal morphology, we employed immunofluorescence (Fig. 2*A*). Firstly, we measured KCC2 expression levels. KCC2 expression was significantly decreased in neurons infected with AAV-Cre, which were identified by the presence of GFP. We observed a 63.4 ± 2.27% and a 54.6 ± 2.50% decrease in the KCC2 fluorescence levels in GFP-positive regions in developing and mature neuronal networks respectively in the AAV-Cre compared with the AAV-GFP control (Fig. 2*B*, DIV3: *p* < 0.001, DIV18: *p* < 0.001, unpaired *t*-test with Welch’s correction).

The expression of the GFP fluorophore allowed us to reconstruct the neuronal morphology of CaMKII-positive pyramidal cells in the presence and absence of KCC2 (Fig. 2*C*). Using Sholl analysis, it was apparent that reducing KCC2 expression did not modify the complexity of proximal dendrites close to the cell body, as there was no morphological difference from the control in either experimental groups (Fig. 2*C*, *p* > 0.99, Kolmogorov–Smirnov (KS) test). Likewise, the number of intersections between the dendritic segments were also unaffected at either time point.

We also tested if reduced KCC2 expression impacted on the formation of dendritic spines. The loss of KCC2 had no robust effect on dendritic spines, as spines were still present in the absence of KCC2 from both developing and mature networks (Fig. 2*D*). Interestingly, a mild reduction in the number of spines (Fig. 2*D*, Control_DIV18_: 46.9 ± 3.07 spines, Cre_DIV18_: 39.6 ± 1.77 spines, *p* = 0.035, unpaired *t*-test) and an increase in stubby structures were observed when KCC2 was removed from mature networks, which could reflect an increase in immature spines. No significant reduction in either gephyrin or PSD95 (inhibitory and excitatory postsynaptic markers respectively) expression was evident in mature neurons deficient in KCC2 (Fig. 2*E*). Collectively these results suggest that KCC2 does not play a major role in regulating neuronal arborization, dendritic structure, or synapse formation.

### Ablating KCC2 expression prevents the development of hyperpolarizing GABA_A_R currents

**Figure 3:**
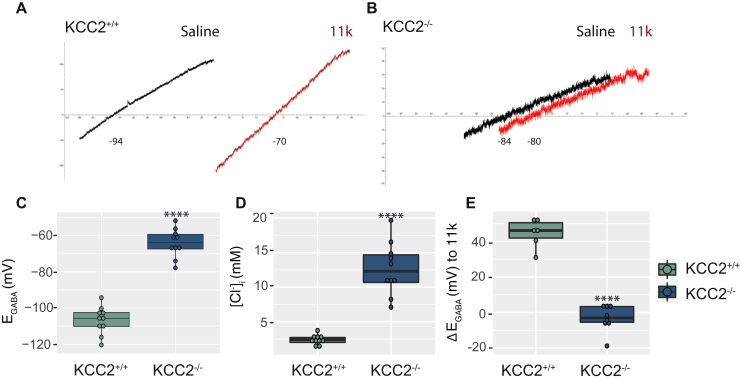
**The effect of KCC2 knockout on E**_**GABA**_**and intracellular Cl**^**−**^**levels.***A*, representative electrophysiological traces of DIV21 neurons infected with AAV-GFP or AAV-Cre at DIV3 showing the leak-subtracted muscimol-activated currents and the shift after 1 μM 11K application for control and *B*, knockout cells. The *box plots* show the quantification for the *C*, mean E_GABA_ (n_KCC2+/+_ = 10, n_KCC2−/−_ = 10 recordings), *D*, calculated Cl^−^ concentration (n_KCC2+/+_ = 10, n_KCC2−/−_ = 10 recordings) and *E*, 11K-induced shift (n_KCC2+/+_ = 6, n_KCC2−/−_ = 8 recordings).

We performed gramicidin perforated-patch recordings on AAV-GFP or AAV-Cre infected neurons to examine the effects of reducing KCC2 expression levels on the reversal potential of GABA-induced currents (E_GABA_), which in mature neurons is predominantly set by KCC2 activity. E_GABA_ was measured by calculating the reversal potential of leak-subtracted muscimol currents (1 μM) during positive-directed voltage ramps. To isolate the contribution of KCC2 to E_GABA_, the NKCC1 inhibitor bumetanide (10 μM) was applied and neuronal activity was blocked using tetrodotoxin (TTX; 500 nM) to prevent activity-dependent Cl^−^ accumulation (Fig. 3, *A* and *B*). Removing KCC2 from developing networks resulted in more depolarized E_GABA_ values in the AAV-Cre infected cells compared with AAV-GFP infected controls, demonstrating the critical role of the KCC2 transporter (Fig. 3*C*, Control_DIV3_: −106.7 ± 2.42 mV, Cre_DIV3_: −64.2 ± 2.52 mV, *p* < 0.001, unpaired *t*-test). This was also reflected as an increase in the intracellular concentrations of Cl^−^ when KCC2 was removed (Fig. 3*D*, Control_DIV3_: 2.4 ± 0.22 mM, Cre_DIV3_: 12.7 ± 1.21 mM, *p* < 0.001, unpaired *t*-test with Welch’s correction). Furthermore, treatment with the selective KCC2 inhibitor, VU0463271 (11K; 1 μM), led to minimal shifts in E_GABA_ values in AAV-Cre infected cells, supporting the absence of KCC2 (Fig. 3*E*, Control_DIV3_: 44.7 ± 3.25 mV, Cre_DIV3_: −3.2 ± 2.69 mV, *p* < 0.001, unpaired *t*-test). In summary, these results are consistent with the well-supported hypothesis that KCC2 expression is critical for the postnatal development of hyperpolarizing GABA_A_R currents.

### Inactivation of KCC2 in mature neurons induces cell death

**Figure 4:**
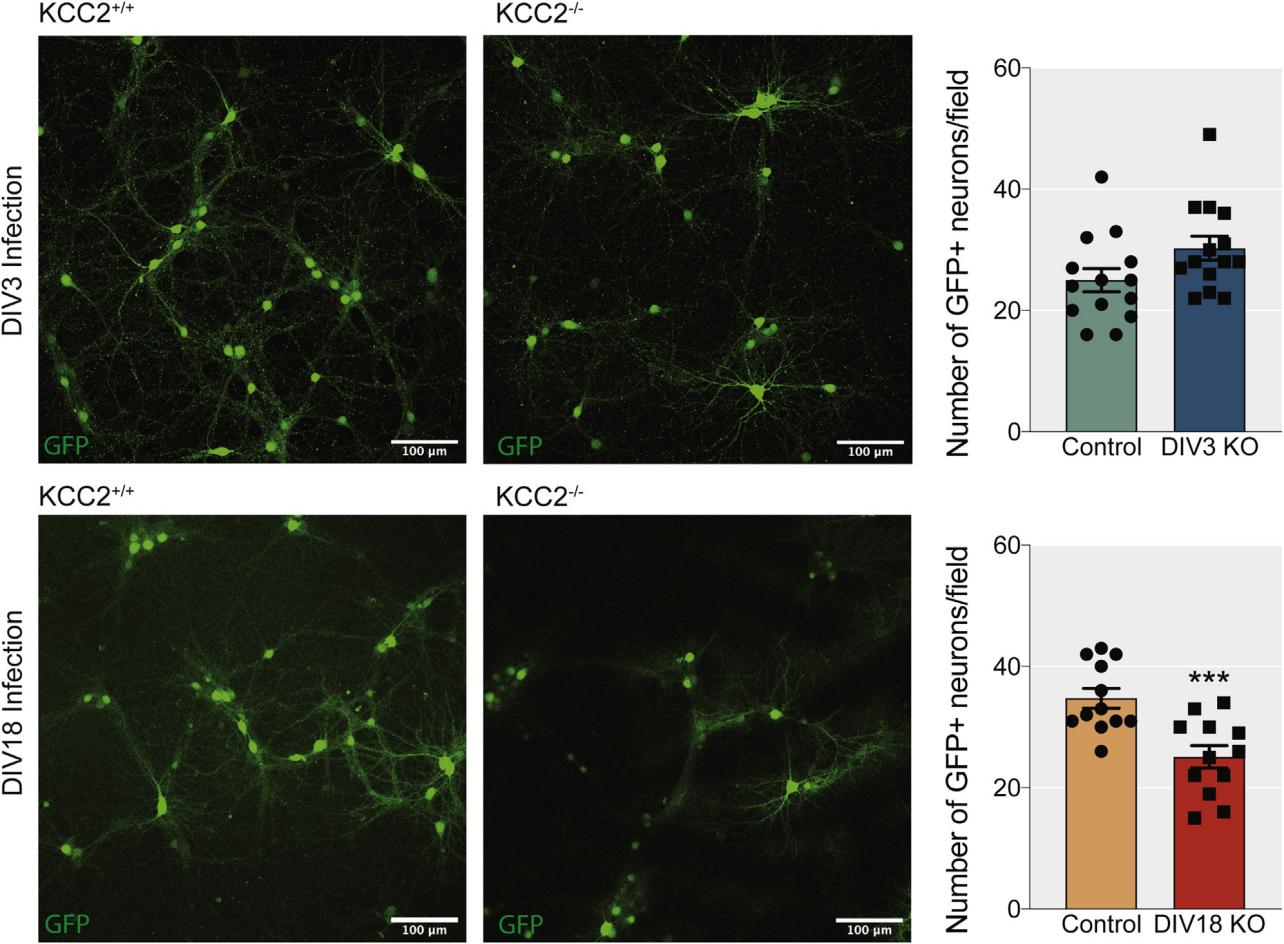
**Loss of KCC2 from mature, but not developing neurons, reduces the number of GFP-expressing neurons in dissociated cultures.** The upper panels show low-magnification 1024 × 1024 confocal images of DIV21 GFP+ neurons in dissociated cultures treated with AAV-GFP and AAV-Cre virus at DIV3 (Scale bar = 100 μm). The bar chart shows the quantification of the number of GFP+ neurons per field of view (Area = 0.405 mm^2^) (n_KCC2+/+_ = 14, n_KCC2−/−_ = 14 images from four individually infected cultures). The lower panels show low-magnification 1024 × 1024 confocal images of DIV24 GFP+ neurons in dissociated cultures treated with AAV-GFP and AAV-Cre virus at DIV18 (Scale bar = 100 μm). The bar chart shows the quantification of the number of GFP+ neurons per field of view (Area = 0.405 mm^2^) (n_KCC2+/+_ = 12, n_KCC2−/−_ = 12 images from four individually infected cultures).

We measured whether the removal of KCC2 in immature and mature neurons affected neuronal viability (Fig. 4). This was carried out by counting the number of GFP-expressing cells in cortical/hippocampal mixed cultures from KCC2^fl^ mice infected at DIV3 or DIV18 with AAV-GFP or AAV-Cre. KCC2 removal did not alter the number of GFP-expressing neurons in DIV3-infected cultures. In contrast, cultures infected at DIV18 showed a significant loss of GFP-positive neurons (Control_DIV18_: 35 ± 1.6 cells, Cre_DIV18_: 25 ± 1.8 cells, *p* = 0.0007, unpaired *t*-test).

### Inactivation of KCC2 in mature neurons induces apoptosis

**Figure 5:**
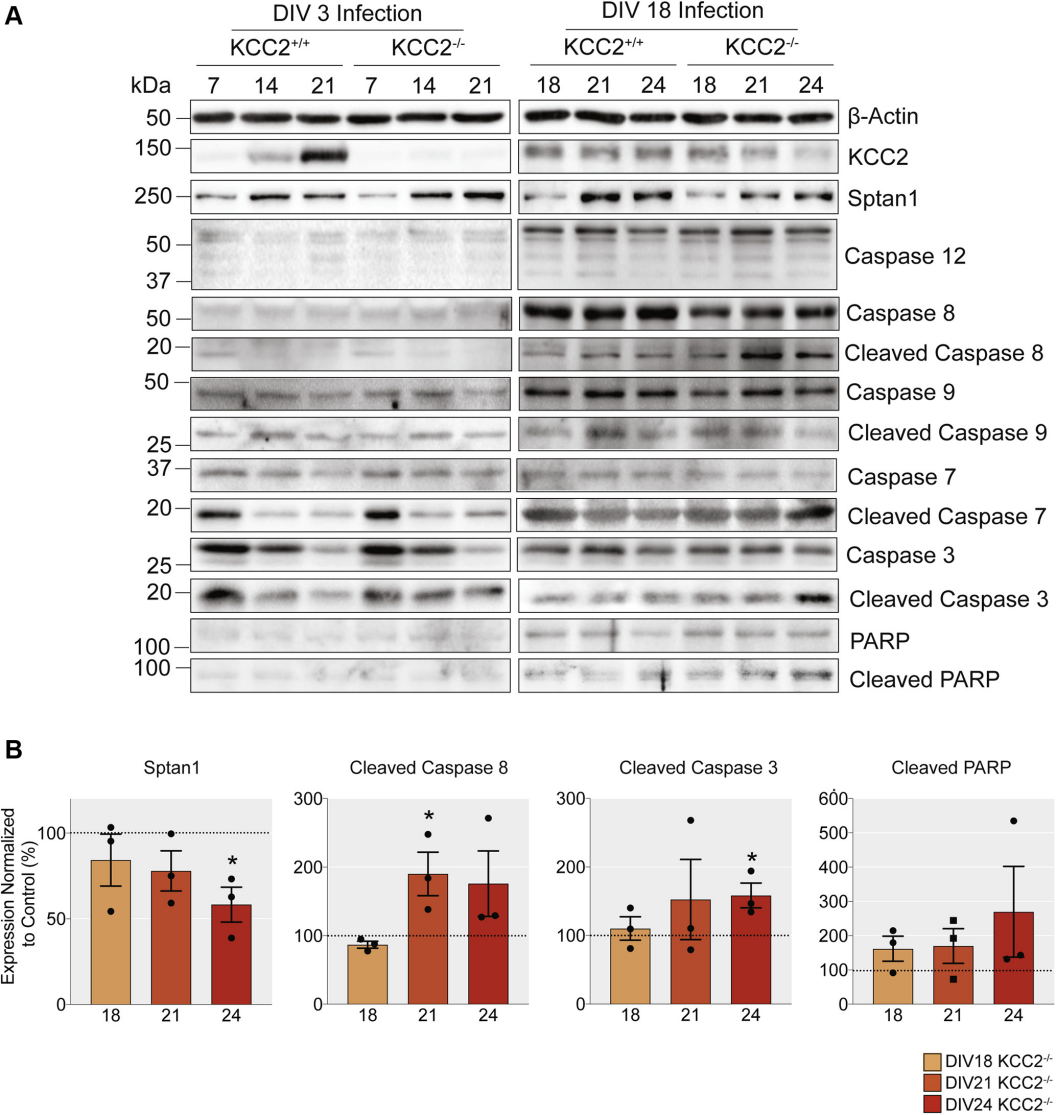
**Reduced KCC2 expression in mature, but not developing neuronal networks, increases the levels of proapoptotic cleaved caspases.***A*, immunoblots on lysates of cortical/hippocampal cultures infected with AAV-GFP or AAV-Cre at DIV3 or DIV18. Lysates were probed for the total and cleaved levels of the extrinsic proapoptotic marker; caspase 8, the intrinsic proapoptotic markers; caspase 3 and caspase 7, the inflammation proapoptotic caspase 12, PARP, KCC2, the KCC2-associated structural protein Sptan1, and β-actin. *B*, removal of KCC2 from mature networks resulted in an increase in the expression of several apoptotic markers. The *bar charts* show the quantification of the levels of cleaved caspase 8, cleaved caspase 3, and Sptan1, with significantly changed expression marked (∗*p* < 0.05, n = 3 replicates).

Work presented here and previously from our lab has shown that a reduction of KCC2 levels decreases the viability of mature hippocampal tissue and cortical/hippocampal cultures (18), with the number of GFP-expressing neurons reduced in dissociated neuronal cultures infected with AAV-Cre at DIV18. In order to understand the mechanism by which KCC2 and disregulated Cl^−^ homeostasis could modulate neuronal viability, we performed western blot analysis and probed neuronal lysates for a range of extensively characterized apoptotic markers, after viral infection with AAV-GFP or AAV-Cre (Fig. 5*A*). We included specific markers that differentiate between the intrinsic and extrinsic apoptotic pathways (20). Under control conditions, the levels of cleaved caspases were low when cells were treated with AAV-GFP at both infection time points (DIV3 and DIV18). In contrast, a 90.1 ± 31.78% increase in the signal of the early proapoptotic marker, cleaved caspase 8, was evident at DIV21, when KCC2 was knocked out at DIV18 (Fig. 5*B*, *p* = 0.047, unpaired *t*-test). The increase in cleaved caspase 8, which mediates the extrinsic pathway of apoptosis, coincides with a loss of KCC2 signal in mature neurons infected with AAV-Cre, compared with the AAV-GFP infected control (Figs. 1*E* and 5*B*). Interestingly, there were no changes in the cleavage of caspases associated specifically with intrinsic pathway activation (caspase 9) when KCC2 was knocked out from either immature or mature neurons. In parallel, a 58.4 ± 18.14% increase in the levels of the late proapoptotic marker; cleaved caspase 3, was seen by DIV24 (Fig. 5*B*, *p* = 0.032, unpaired *t*-test). Thus, the sustained absence of KCC2 by DIV24 resulted in increased levels of active apoptotic executioner caspases, which lead to cell death *via* the activation of the extrinsic apoptotic pathway. In the brain, KCC2 is associated with Sptan1 in large macromolecular complexes (21). The levels of Sptan1, a previously characterized calpain and caspase 3 substrate and marker of neuronal damage (22, 23), were decreased to 58.3 ± 10.20% by DIV24 in the absence of KCC2 (Fig. 5*B*, *p* = 0.015, unpaired *t*-test). In contrast, there were no changes in the levels of apoptotic markers in cells infected with AAV-Cre at DIV3, suggesting that KCC2 removal from developing networks does not impact neuronal viability. Thus, our results suggest that in mature but not developing neuronal networks, loss of KCC2 induces apoptotic cell death.

### Acute pharmacological inhibition of KCC2 induces apoptosis

**Figure 6:**
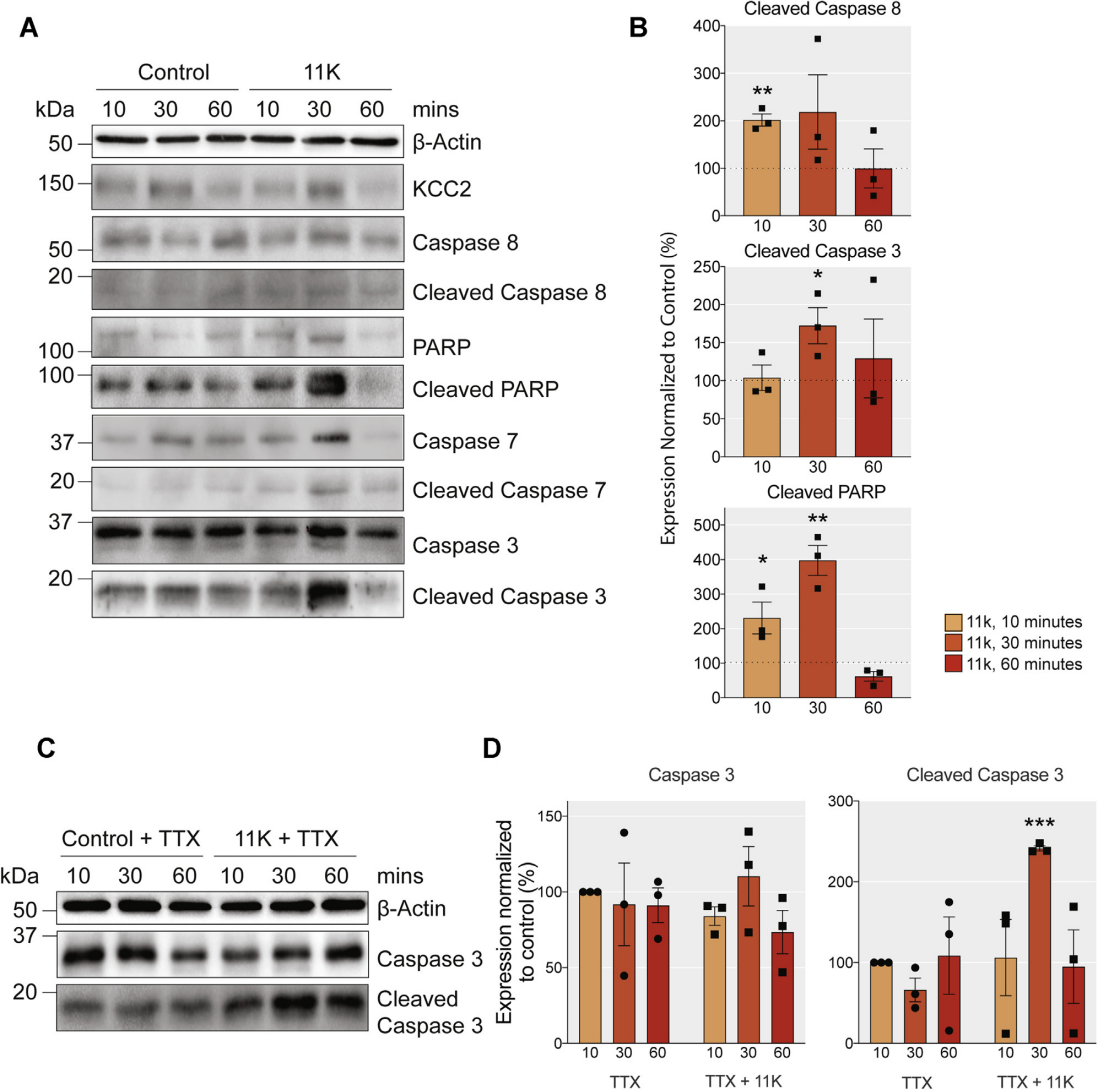
**Pharmacological inhibition of KCC2 with 11K stimulates apoptosis in neuronal cultures.***A*, immunoblots of DIV18-DIV21 cortical/hippocampal neuronal lysates treated with DMSO or 1 μM 11K for 10, 30, or 60 min were probed for the total and cleaved expression of the extrinsic proapoptotic marker; caspase 8, the intrinsic proapoptotic markers; caspase 3 and caspase 7, PARP, KCC2, and β-actin. *B*, the *bar charts* show the quantification of the levels of proapoptotic markers cleaved caspase 8, cleaved caspase 3, and cleaved PARP, with significantly changed expression marked (∗*p* < 0.05, ∗∗*p* < 0.01, n = 3 replicates). *C*, immunoblots of DIV18–21 cortical/hippocampal neuronal lysates with 500 nM TTX alone or TTX+11K for 10, 30, or 60 min were probed for expression of total and cleaved caspase 3. *D*, the *bar charts* show the quantification of total and cleaved caspase 3 expression.

To investigate the role that KCC2 activity plays as a determinant of neuronal viability further, we examined the effects of its acute inhibition on apoptotic markers. To do so, DIV18–21 cultured neurons were exposed to the selective KCC2 inhibitor; 11K or DMSO (control) for 10, 30, and 60 min, and subsequently lysed and subjected to immunoblotting (Fig. 6, *A* and *B*). Exposure to 11K increased the level of the early apoptotic marker; cleaved caspase 8 by 101.7 ± 12.84% compared with the control, within 10 min (*p* = 0.001, unpaired *t*-test). This was followed at 30 min by a 72.5 ± 23.78% increase in the late apoptotic marker: cleaved caspase 3 (*p* = 0.038, unpaired *t*-test). Furthermore, we also observed a striking fourfold increase in the levels of cleaved poly (ADP-ribose) polymerase (PARP) (*p* = 0.002, unpaired *t*-test). Thus, acute inhibition of KCC2 is sufficient to activate the extrinsic apoptotic cascade. Next, we repeated the experiment in the presence of TTX to study whether this induction of apoptosis was dependent on neuronal activity (Fig. 6, *C* and *D*). TTX did not prevent the cleavage of caspase 3 induced by 11K (Fig. 6*D*, *p* = 0.0003, unpaired *t*-test).

### Local inactivation of KCC2 in the hippocampus induces apoptosis

**Figure 7:**
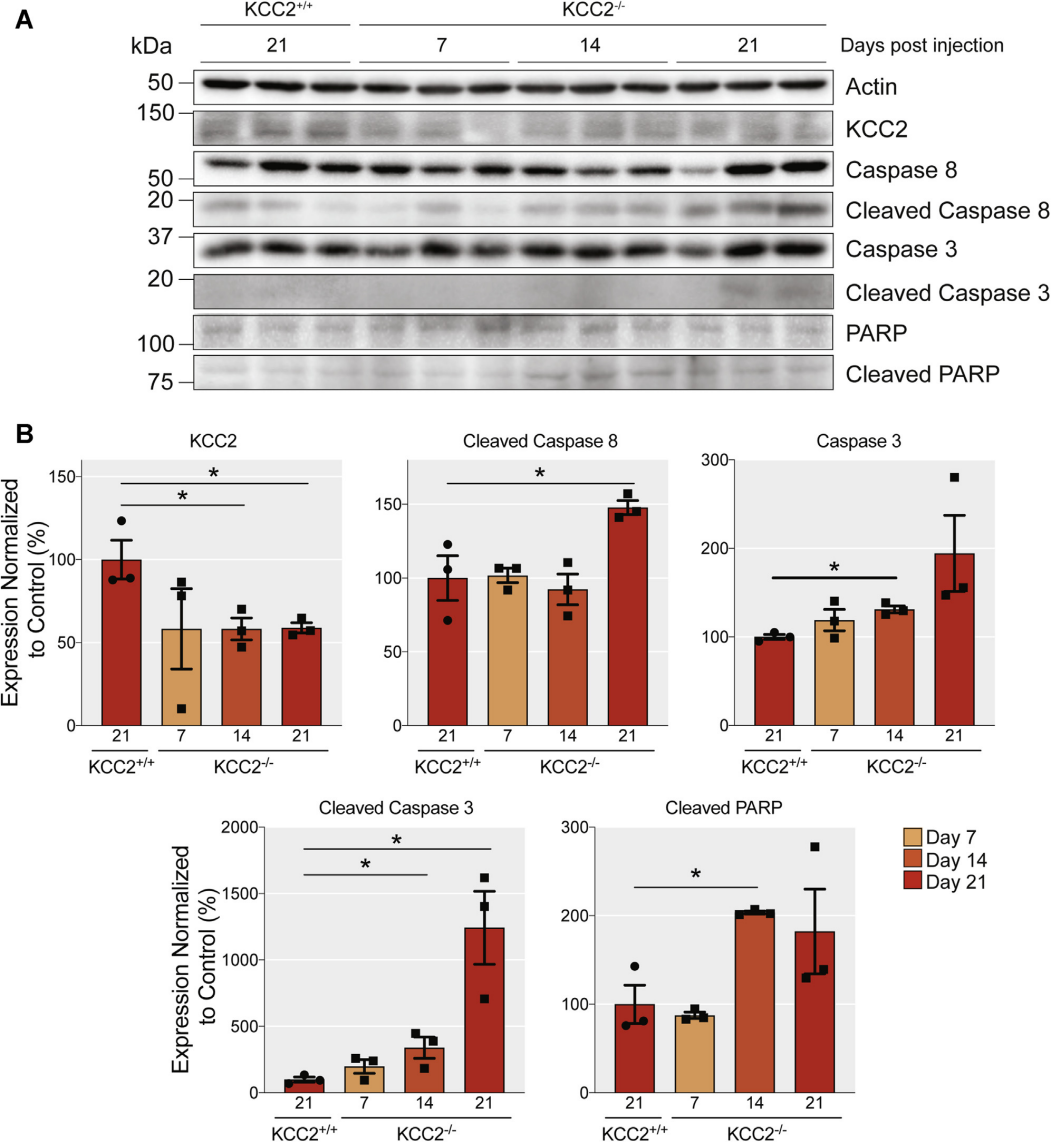
**Loss of KCC2 *in vivo* increases the levels of proapoptotic markers.***A*, immunoblots of cortical/hippocampal brain lysates harvested 7, 14, and 21 days after viral infection with AAV-GFP or AAV-Cre probed for the total and cleaved levels of the extrinsic proapoptotic marker caspase 8, the intrinsic proapoptotic markers caspase 3 and caspase 7, the DNA damage marker PARP, KCC2, and β-actin. *B*, the *bar chart* shows the quantification of the levels of proapoptotic markers cleaved caspase 8, cleaved caspase 3, and cleaved PARP, with significantly changed expression marked (∗*p* < 0.05, n = 3 replicates).

To assess the significance of our results in cultured neurons, we looked at the effects of reducing KCC2 expression on apoptotic markers in the brain. To do so, stereotaxic injections were used to introduce AAV-GFP or AAV-Cre, into the hippocampus of 8- to 12-week-old KCC2^fl^ mice. The brains were harvested 7-, 14-, and 21 days following injection as detailed by Kelley *et al.*, 2018. This approach led to significantly reduced KCC2 expression in the CA1 region and the dentate gyrus. In addition, this inactivation led to the death of neurons in both hippocampal subdomains and increased gliosis 28 days after injection. To provide insights into the mechanism of cell death, infected tissue was identified *via* GFP expression and microdissected under a fluorescence microscope. The extracted tissue was then lysed for western blot analysis, and immunoblotting was used to measure the levels of apoptotic markers between AAV-GFP and AAV-Cre infected groups (Fig. 7). Consistent with previous data, infection with the AAV-Cre was sufficient to reduce the expression of KCC2 in mouse hippocampal neurons. The total KCC2 levels dropped to 59 ± 3.1% by 21 days after the AAV-Cre injection (*p* = 0.027, unpaired *t*-test). We observed a 47.8 ± 4.79% increase in the protein levels of cleaved caspase 8 in the brains injected with AAV-Cre when compared with the AAV-GFP control, 21 days after injection (*p* = 0.04) and a striking tenfold increase in the levels of cleaved caspase 3 (*p* = 0.014, unpaired *t*-test), 21 days after injection with AAV-Cre along with a significant increase in the expression of cleaved PARP 14 days after injection (*p* = 0.009, unpaired *t*-test). Thus, removal of KCC2 is also sufficient to induce apoptosis in the brain.

## Discussion

KCC2 provides the principle Cl^−^ extrusion mechanism for cells in the CNS. Here we used the previously described KCC2^fl^ mouse (19) combined with Cre-expressing AAVs to achieve highly efficient conditional removal of KCC2 in excitatory neurons (Fig. 1). Using this strategy, we have begun to explore the role that KCC2 plays in determining neuronal morphology, neuronal survival, and the postnatal development of GABAergic inhibition in immature and mature neuronal networks. The placement of the *loxP* sites in the *SLC12A5* gene sequence of the KCC2^fl^ mouse should result in a protein lacking the protein sequence flanked by Q911-Q1096. However, the data presented here compliments previous observations that truncation of the *SLC12A5* gene results in the ablation of KCC2 expression (18). This was demonstrated by immunoblotting with N- and C- terminal antibodies and suggests that either the truncated form of KCC2 protein is unstable and does not accumulate or the altered gene sequence results in premature transcriptional or translational termination. This is contrary to previous work using truncated KCC2 constructs (9, 10) and likely represents the differences associated with endogenous *versus* overexpression systems. We were not able to completely ablate KCC2 expression *in vitro* or *in vivo* under these experimental conditions. When KCC2 was removed from immature networks (DIV3 infected), which initially express low levels of KCC2, the residual KCC2 detected is likely expressed by interneurons (24). In mature networks, however (DIV18 infected), where the KCC2 expression level is initially much higher, the residual KCC2 observed is likely to be a combination of interneuron expression and the result of slow protein turnover or local translation of slow turnover mRNAs. This is consistent with previous work estimating that the half-life of KCC2 can be in the order of days (25).

Our approach aimed to differentiate between the removal of KCC2 in immature networks compared to mature networks and study the resulting impact on neuronal morphology, activity, and viability *in vitro*. The methodology used in our *in vitro* experiments enabled us to examine the effects of KCC2 removal with high temporal precision, which cannot be attained *in vivo*, as mice deficient in this transporter die shortly after birth (26). The work presented here compliments the recent study that demonstrated that the genetic removal of KCC2 during neuronal migration induces the apoptosis of embryonic neocortical projection neurons (27). Yet, the functional consequences of KCC2 removal during postnatal neuronal development have not been investigated and are of great interest as a number of studies have suggested that KCC2 plays a transport-independent role in determining spine morphology (9, 10). These studies used overexpression of a KCC2 construct devoid of its cytoplasmic tail (residues Q911–Q1096) or a DNA construct modified with a GFP reporter, which may act to stabilize this truncated form of KCC2 (9, 10).

The ablation of KCC2 expression at DIV3 had no impact on the subsequent development of neurons (Fig. 2). Both the complexity of proximal dendritic processes and spine formation were comparable with control cells. Likewise, ablating KCC2 expression in immature neurons did not induce cell death (Fig. 4) or apoptosis, as measured by cleaved caspases 3, 7, 8, or PARP, to detect apoptosis (Fig. 5). However, hyperpolarizing GABA_A_R currents were compromised in neurons with reduced KCC2 expression (Fig. 3) (18), despite unchanged expression of the inhibitory synapse scaffold protein; gephyrin. Thus, the presence of KCC2 constitutes a prerequisite for GABAergic inhibition but may not contribute to neuronal morphological development or viability. Therefore, our results further highlight the critical role of KCC2 in the postnatal development of GABAergic inhibition, a process that has been proposed to be mediated in part by impermeant anions (28).

While the removal of KCC2 at DIV3 mimics the loss of KCC2 during postnatal development, the removal of KCC2 at DIV18, from mature networks that exhibit KCC2 activity-dependent hyperpolarizing GABA_A_R currents (1, 18), mimics the acute loss of KCC2 following ischemic injury or seizures (12, 29). Such acute neuronal injuries can cause gross neuronal damage, including alterations in spine morphology (30), and initiate the neuroinflammatory response, ultimately resulting in cell death (31). Previous work has demonstrated that conditional knockout of KCC2 in the adult mouse hippocampus induced seizures and neuronal loss (18). Here, following 6 days of viral exposure initiated at DIV18, we observed subtle changes in spine maturity consistent with previous observations (Fig. 2) (9, 10). However, at this time point, we also detected a significant reduction in the number of GFP-positive neurons (Fig. 4), indicating cell death, and multiple markers of apoptotic induction (Fig. 5). Ablating KCC2 expression in mature cultures resulted in a significant increase in cleaved caspase 8 after 3 days of viral exposure. Caspase 8 is part of the extrinsic apoptotic pathway and is cleaved in response to extracellular signals. For instance, proteins such as tumor necrosis factor α (TNF-α) and tumor necrosis factor–related apoptosis-inducing ligand (TRAIL) bind to cognate death receptors, localized in the target cell plasma membrane (20, 32). In the brain, these proteins are released in response to neuronal injury (33, 34). The cleavage of caspase 8 can result in both initiating the downstream caspase cascade leading to apoptosis or perpetuating the inflammatory response (35) and has been demonstrated in seizure-induced apoptotic cell death (36, 37). One of the immediate downstream consequences of caspase 8 cleavage is calpain activation, which in turn cleaves numerous protein targets. Sptan1 is a KCC2-associated protein (21) and is cleaved by calpains and caspases in response to neuronal injury (38) in a similar manner to KCC2 itself (25). After 6 days of viral exposure, loss of full-length Sptan1 was observed, as well as cleaved downstream proteins; caspases 3, 7, and PARP. Cleavage of these proteins is indicative of the final stages of apoptotic cell death (39). Consistent with our experiments *in vitro* locally reducing KCC2 expression in the hippocampus of adult mice was sufficient to activate the extrinsic pathway of apoptosis.

In addition to KCC2 gene knockout, we assessed the effects of pharmacological inhibition of KCC2 on apoptosis using the specific KCC2 inhibitor; 11K (40). *In vitro* blockade of KCC2 induced caspase 8, 3, and PARP cleavage within 30 min. Previous studies have shown that consistent with KCC2 inhibition, 11K induces rapid neuronal depolarization and seizure-like events (40, 41, 42). Significant inhibition of neuronal depolarization with TTX did not impair the effects of 11K on caspase activation, strongly suggesting that it is independent of neuronal activity. The rapid induction of apoptosis by 11K *via* the extrinsic pathway observed here in 10 to 30 min is highly consistent with that observed in cell lines (43) and that observed following ischemic injury (44).

Taken together, these results indicate that removal of KCC2 from mature neuronal networks causes changes in spine morphology; however, this may at least be due to the induction of apoptosis *via* the extrinsic pathway. Data presented here suggests that changes in spine morphology, following KCC2 removal, are due to an apoptotic event and impact the notion that KCC2 has transporter-independent functions.

Here, we have developed an elegant method of studying immature and mature neuronal networks in the presence or absence of KCC2. Our results demonstrate that reducing KCC2 expression levels in immature neurons does not impact gross neuronal morphology or viability but prevents the development of GABAergic inhibition. In contrast, mature neuronal networks are highly sensitive to KCC2 removal, where a reduction of approximately 50% is sufficient to induce apoptosis. This work advances our understanding of the pathological mechanisms that proceed reduced KCC2 expression and suggests that restoring KCC2 activity following traumatic events such as seizures or ischemia may prevent neuronal loss by apoptosis.

## Experimental proceedures

### Animal Care

Animal studies were performed according to protocols approved by the Institutional Animal Care and Use Committee of Tufts Medical Center (IACUC). Mice were kept on a 12 h light/dark cycle with *ad libitum* access to food and water.

### Antibodies

The primary antibodies used in this study were: KCC2 (mouse, Neuromab 75-013, WB 1:1000, ICC 1:500–1000), KCC2 (rabbit, LSBio LS-C135150, WB 1:1000), KCC2 (rabbit, Millipore 07-432, ICC 1:500–1000), GFP (chicken, Abcam 13970, ICC 1:1000), Caspase 3 (rabbit, CST #9662, WB 1:1000), Cleaved Caspase 3 (rabbit mAb, CST #9664, WB 1:1000), Caspase 7 (rabbit mAb, CST #12827, WB 1:1000), Cleaved Caspase 7 (rabbit mAb, CST #8438, WB 1:1000), Caspase 8 (rabbit mAb, CST #4790, WB 1:1000), Cleaved Caspase 8 (rabbit mAb, CST #8592, WB 1:1000), Caspase 9 (mouse, CST #9508, WB 1:1000), Cleaved Caspase 9 (rabbit mAb, CST #52873, WB 1:1000), Caspase 12 (rabbit, CST #2202, WB 1:1000), PARP (rabbit, CST #9542, WB 1:1000), Cleaved PARP (rabbit mAb, CST #5625, WB 1:1000), Sptan1 (mouse, Abcam 11755, WB 1:1000) and β-actin (mouse, Sigma A1978, WB 1:5000). The secondary antibodies used were: goat anti-chicken Alexa Fluor 488 (Thermo Fisher, 1:1000), goat anti-rabbit Alexa Fluor 555 (Thermo Fisher, 1:1000), goat anti-rabbit Alexa Fluor 568 (Thermo Fisher, 1:1000), goat anti-rabbit Alexa Fluor 647 (Thermo Fisher, 1:1000), donkey anti-rabbit conjugated HRP (Jackson ImmunoResearch, 1:5000), and donkey anti-mouse conjugated HRP (Jackson ImmunoResearch, 1:5000).

### Electrophysiology

Electrophysiological experiments were carried out as previously described (18). Briefly, neurons were recorded at 33 °C in saline containing 140 mM NaCl, 2.5 mM KCl, 2.5 mM MgCl_2_, 2.5 mM CaCl_2_, 10 mM HEPES, 11 mM glucose, pH 7.4 (NaOH). All solutions were applied through three-barrel microperfusion apparatus (700 μm, Warner Instruments) and conducted in the presence of tetrodotoxin (500 nM) and bumetanide (10 μM) to block voltage-dependent sodium channels and NKCC1, respectively. Cells infected with AAV9-CaMKII-eGFP (AAV-GFP), or AAV9-CaMKII-eGFP-Cre (AAV-Cre) (University of Pennsylvania Vector Core) were identified based on GFP expression using an epifluorescence microscope. Data were acquired using Clampex 10 software at an acquisition rate of 10 kHz with an Axopatch 2B amplifier and digitized with a Digidata 1440 (Molecular Devices). To detect endogenous Cl^−^ levels, cells were perforated with gramicidin (50 mg/ml) dissolved in an internal pipette containing 140 mM KCl, 10 mM HEPES, pH 7.4 (KOH), and a tip resistance of 3 to 4 MΩ. Following perforation to below a series resistance of 100 MΩ, E_GABA_ values were detected by application of muscimol (1 μM) during positive-going voltage ramps (20 mV/s).

### Generation and genotyping of KCC2^fl^ mice

KCC2^fl^ mice (*Slc12a5*lox/lox) were described previously (18, 19) and have been backcrossed on the C57BL/6 J background for at least ten generations.

### Image analysis

For immunoblots, individual band intensity was quantified using densitometry on Fiji (Version 1.0), normalized to β-actin, and further normalized to the corresponding control condition where appropriate. For immunocytochemistry (ICC), the KCC2 fluorescent intensity in 1024 × 1024 confocal images was quantified on Fiji and normalized to the control (45). Briefly, a mask was generated manually or using the thresholded GFP signal, which was then superimposed on the KCC2 fluorescent channel to measure the signal within the GFP-positive region. The GFP signal was also used to reconstruct proximal dendrites manually, generate a .SWC file, and perform the Sholl analysis using the Simple Neurite Tracer Plugin on Fiji (46).

### Immunoblotting

Immunoblotting was carried out as previously described (34). For cultured cortical/hippocampal neurons, media was removed and spun down at 1000 g for 5 min to harvest potentially apoptotic floating cells. Adherent cells were washed twice with phosphate-buffered saline (PBS), combined with floating cells, and lysed in RIPA buffer containing proteases and phosphatase inhibitors. For wild-type cultured neurons treated with TTX/11K, cells were washed in PBS and lysed in RIPA buffer containing proteases and phosphatase inhibitors. For tissue, GFP-positive regions of the hippocampus infected with AAV-GFP or AAV-Cre were microdissected under a fluorescent microscope. The tissue was homogenized in RIPA buffer containing protease and phosphatase inhibitors. Proteins were quantified by Bradford assay. Samples were diluted to the same concentration in RIPA buffer and 2X sample buffer added. Samples were boiled for 5 min at 95 °C before being loaded onto polyacrylamide gels for sodium dodecyl sulfate–poly acrylamide gel electrophoresis (SDS-PAGE). Proteins were then transferred onto nitrocellulose membranes, blocked in milk for 1 h, and probed with primary antibodies overnight. The membranes were washed and probed with appropriate HRP-conjugated secondary antibodies and developed with enhanced chemiluminescent (ECL) substrate in a Bio-Rad ChemiDoc Imager. Where possible all replicates were run on the same gel.

### Immunocytochemistry

ICC was carried out as previously described (21). Briefly, primary cultured cortical/hippocampal neurons were washed with PBS, fixed in 4% paraformaldehyde (PFA, Electron Microscopy Services) in PBS, and permeabilized in block solution consisting of 1X PBS with 0.1% Triton X-100, 3% (w/v) Bovine Serum Albumin (BSA), 10% normal goat serum, and 0.2 M glycine. Primary and secondary antibodies (as described above) were prepared at a dilution of 1:1000 in block solution and incubated with the cells for 1 h at room temperature in the dark. The cells were washed in 1X PBS and mounted on glass slides using ProLong Gold.

### Primary neuronal culture

Primary cortical/hippocampal mixed neuronal cultures were prepared and cultured as previously described (47). Briefly, P0 KCC2^fl^ mice were anesthetized on ice and the brains were removed. The brains were dissected in Hank's buffered salt solution (HBSS, Invitrogen) with 10 mM HEPES. The cortices and hippocampi were trypsinized and triturated to dissociate the neurons. Cells were counted using a hemocytometer and plated on poly-l-lysine-coated coverslips (for ICC and electrophysiology) or in 35 mm dishes (for immunoblot) at a density of 1 × 10^5^ or 4 × 10^5^ cells respectively. At days *in vitro* (DIV) 3 or 18, cells were exposed to AAV-GFP or AAV-Cre as described above at a concentration of 1 × 10^6^ genome copies (GC)/ml. For biochemical analysis and ICC, cells infected at DIV3 were harvested at DIV7, 14, and 21 and cells infected at DIV18 were harvested at DIV18, DIV21, and DIV24. At DIV18–21, primary neurons were treated with either DMSO or 11K (1 μM) for 10, 30, and 60 min before being harvested for biochemical analysis.

### Statistics

Fluorescent imaging and perforated patch-clamp data were subjected to the D’Agostino–Pearson omnibus normality test and the F-test to compare variances on GraphPad Prism (Version 9.0). To assess statistical significance, the unpaired two-sample *t*-test was performed, and the Welch’s correction was applied when necessary. The cumulative distributions generated from Sholl analysis were subjected to the KS test. To assess statistical significance, the western blot data were subjected to unpaired two-sample *t*-test. Significance of *p* < 0.05 is represented as ∗, *p* < 0.01 is represented as ∗∗, *p* < 0.001 is represented as ∗∗∗, and *p* < 0.0001 is represented as ∗∗∗∗.

### Stereotaxic injection

Stereotaxic injection was carried out as previously described (18). Briefly, age-matched male WT and KCC2^fl^ animals (8–12 weeks) were bilaterally injected with AAV expressing Cre recombinase. Prior to surgery, animals were anesthetized by isoflurane inhalation and administered buprenorphine HCl (0.1 mg/kg) subcutaneously as analgesic. The deeply anesthetized animals were immobilized and stabilized in a Kopf stereotaxic apparatus. Holes were drilled on the skull surface, and Neurosyringe (Hamilton) was used to deliver 1 μl of AAV-GFP or AAV-Cre (1 × 10^13^ GC/ml) into each of the CA1 areas with the following coordinates relative to Bregma: AP: −2.0, ML: ±1.8, DV: −2.0. AAV was injected at a rate of 100 nl/min, under the control of a microsyringe pump controller (World Precision Instrument Micro4). After the injection, the incision site was sutured, and animals were housed individually and postoperatively monitored.

## Data availability

All data are contained within the article.

## Conflict of interest

S. J. M. serves as a consultant for AstraZeneca and SAGE Therapeutics, relationships that are regulated by Tufts University. S. J. M. holds stock in SAGE Therapeutics.
